# Characteristics of Toxic Keratopathy, an *In Vivo* Confocal Microscopy Study

**DOI:** 10.1167/tvst.10.11.11

**Published:** 2021-09-08

**Authors:** Leying Wang, Yang Zhang, Zhenyu Wei, Kai Cao, Guanyu Su, Pedram Hamrah, Antoine Labbe, Qingfeng Liang

**Affiliations:** 1 Beijing Institute of Ophthalmology, Beijing Tongren Eye Center, Beijing Key Laboratory of Ophthalmology and Visual Sciences, Beijing Tongren Hospital, Capital Medical University, Beijing, China; 2 Cornea Service, New England Eye Center, Center for Translational Ocular Immunology, Department of Ophthalmology, Tufts Medical Center, Tufts University School of Medicine, Boston, MA, USA; 3 Quinze-Vingts National Ophthalmology Hospital, IHU FOReSIGHT, Paris and Versailles Saint-Quentin-en-Yvelines University, Versailles, France; 4 Sorbonne Université, INSERM, CNRS, Institut de la Vision, Paris, France

**Keywords:** dendritiform cell, in vivo confocal microscopy, subbasal nerve, toxic keratopathy

## Abstract

**Purpose:**

Toxic keratopathy (TK) involves complex clinical manifestations and is difficult to differentiate from other ocular surface diseases by conventional slit-lamp examination. The challenge faced by clinicians in confidently diagnosing TK cannot be underestimated. This study aimed to explore the microstructural characteristics and diagnostic parameters by in vivo confocal microscopy (IVCM) in TK.

**Methods:**

In this prospective, cross-sectional, comparative study, slit-lamp and IVCM examinations were performed on 20 normal eyes and 54 eyes with TK. Based on slit-lamp imaging, TK subjects were divided into four groups: superficial punctate keratitis (*n* = 10 eyes), pseudodendritic keratitis (*n* = 14 eyes), ulcerative keratitis (UK; *n* = 16 eyes), and ring keratitis (RK; *n* = 14 eyes). The microstructural characteristics of TK were described according to the following IVCM parameters: basal cell (BC) density, dendritiform cell (DC) density, DC size, corneal nerve fiber (CNF) length, nerve tortuosity, and keratocyte reflectivity. A receiver operating characteristic (ROC) curve model was also formulated to compare the predictive power of BC density, DC density, and CNF length.

**Results:**

TK eyes showed significantly higher values for DC density (45.8 cells/mm^2^; range, 25.0–100.0) compared with normal eyes (24.0 cells/mm^2^; range, 20.8–32.3; *P* = 0.013; DC size (111.0 µm^2^; range, 92.0–137.8) compared with normal eyes (63.7 µm^2^; range, 47.7–70.3; *P* < 0.001); nerve tortuosity (0.08; range, 0.05–0.09) compared with normal eyes (0.04; range, 0.02–0.04; *P* < 0.001); and keratocyte reflectivity. BC density and CNF length values were found to be significantly less than those for normal controls (both *P* < 0.001). In all subgroups, CNF length appeared to be significantly lower than that of controls (all *P* < 0.001), and DC density was only statistically significantly higher in the UK (*P* = 0.003) and RK (*P* < 0.001) groups. Corneal fluorescein staining had no correlation with the analyzed IVCM parameters (all *P ˃* 0.05). However, the increase in DC density and DC size showed negative correlations to CNF length (density: *r* = −0.325, *P* < 0.005; size: *r* = −0.493, *P* < 0.005), as well as positive correlations to duration and frequency of topical eye drops and DC size (density: *r* = 0.361, *P* < 0.05; size: *r* = 0.581, *P* < 0.05). A ROC curve showed that CNF length had the strongest predictive power, with the estimated area under the curve being 0.992 ± 0.008.

**Conclusions:**

Lower BC density and CNF length, greater DC density and DC size, and greater keratocyte reflectivity were the microstructural characteristics of TK. The role of subbasal nerve, inflammatory response, and limbal stem cells in the progression of TK and the appropriate treatment of different TK stages are future research directions.

**Translational Relevance:**

The evaluation of basal cells, subbasal nerve, and dendritiform cells is helpful to our understanding of the pathological process of TK.

## Introduction

Toxic keratopathy (TK) is defined as corneal pathological changes or a functional disturbance with or without an inflammatory response, caused by topical or systemic medications and associated preservatives or breakdown products of the drug.^1^ Due to its potentially sight-threatening nature, TK must be recognized and managed correctly.^2^^,^^3^ However, TK has no specific symptoms or signs, and it is difficult to distinguish it from other corneal conditions, especially chronic ocular irritation or persistent epithelial erosions.^4^^,^^5^ Misdiagnosis of TK results in sustained or increased use of medication, leading to a vicious cycle that can potentially threaten vision and even cause corneal perforation. Timely diagnosis and medication adjustment play an important role in the prognosis of subjects with TK.

TK involves a broad range of presentations from mild punctate or pseudodendritic keratitis to severe or ring ulcerative keratitis.^3^^,^^6^ Superficial punctate keratitis (SPK) is caused by a wide spectrum of ocular surface diseases, such as dry eye disease and Thygeson's superficial punctate keratitis.^7^^,^^8^ Pseudodendritic keratitis (PDK) is difficult to differentiate from viral keratitis.^9^ Ulcerative keratitis (UK) is also a sign of neurotrophic keratopathy, infectious keratitis, and severe blepharitis,^10^^,^^11^ and ring keratitis (RK) is usually associated with *Acanthamoeba* keratitis.^12^ Hence, diagnosis solely based on slit-lamp examination is insufficient, and the diagnosis of TK poses a great challenge for ophthalmologists.

Meanwhile, guidelines for TK therapy have not been established to date except for discontinuation of medications when possible. It is still unclear which stages of TK call for a combination of antiinflammatory drugs and/or neuroregenerative treatment. This is mainly due to the limited in vivo human studies focusing on the cellular changes and differences between each TK subtype. Numerous basic studies have concluded that several eye drops can reduce cellular viability and induce proinflammatory and apoptotic responses in limbal stem cells and trigeminal neuronal cells.^13^^–^^16^ The majority of clinical research on TK has been in the form of case reports or retrospective studies summarizing clinical features.^4^^,^^17^^,^^18^ Only one study has focused on differential diagnosis of toxic SPK at the epithelial cellular level.^7^ However, the changes in dendritiform cells (DCs) and the characteristics of other subtypes of TK have not been thoroughly explored. Thus, a deeper understanding of cellular changes in the various clinical manifestations of TK should be developed in order to guide drug selection.

As an alternative, in vivo confocal microscopy (IVCM), a non-invasive image acquisition method, is capable of providing high-resolution images to evaluate various microstructural changes in the ocular surface under normal and pathologic conditions.^19^^–^^21^ In this study, we used IVCM to assess the quantity and function of limbal stem cells, inflammatory response, subbasal nerve changes, and depth of involvement in various TK subgroups. By combining slit-lamp and IVCM examination, we aimed to explore newly described microstructural characteristics to further enhance the diagnosis and treatment of TK.

## Materials and Methods

### Subjects

This prospective, cross-sectional, comparative study was conducted at the Beijing Tongren Hospital from June 2018 to December 2019, with the approval of the Medical Ethics Committee of Beijing Tongren Hospital (TRECKY2018-129). All participants were informed of the purpose of the study, and their consent was obtained in accordance with the tenets of the Declaration of Helsinki.

Patients who met the following criteria were diagnosed as TK patients: (1) symptoms and signs of ocular surface disease with the use of three or more types of eye drops for over 1 month; (2) slit-lamp examination showing conjunctival congestion, swollen corneal epithelium, diffuse positive fluorescein staining keratopathy, or non-purulent ulcer; (3) IVCM not detecting any fungus or amoeba-like structure; (4) negative corneal smear and microbial culture; and (5) improvement of clinical symptoms and signs 1 week after discontinuation of the original medications and instillation of preservative-free artificial tears.^7^ The included TK subjects were further subdivided into four groups (SPK, PDK, RK, and UK) based on slit-lamp presentation. SPK was defined as superficial, coarse, widespread epithelial keratopathy.^6^^,^^7^ PDK was defined as opaque epithelium in a horizontal “Y” or double-ended “Y” configuration, and the peripheral epithelium in PDK was more coarse, with diffuse punctate staining.^6^ UK was defined as sterile epithelial defects with fluorescein pooling, including pseudogeographic defects and non-pseudogeographic defects, accompanying severe punctate staining in the inferonasal and inferior areas of the cornea.^4^^,^^6^ If a partial or complete ring-shaped infiltrate was noted, the affected eye was included in the RK group.^18^ Subjects with a history of systemic treatment that might affect the cornea, such as amiodarone or phenothiazole, 3 months prior were excluded.^17^^,^^22^

Age- and sex-matched reference controls were healthy, asymptomatic subjects. They had no history of eye disease and were free of any ocular surface abnormalities as determined by slit-lamp examination, tear-film break-up time > 10 seconds, and Schirmer's I test > 10 mm. Control subjects with a history of contact lens wear (consistent wear over 2 weeks) and/or any ocular surface surgery were excluded.^7^

### Clinical Assessment

After the collection of information, including age, sex, symptoms, initial diagnosis, and medication history (drug, frequency, duration), patients underwent slit-lamp examination (Topcon SL-D7; Topcon, Tokyo, Japan). The cornea was divided into five regions: central, superior, inferior, nasal, and temporal. The amount of involvement was evaluated with fluorescein staining (Oxford scale) in each region individually. For corneal defects, the area of the lesion was measured manually with ImageJ software (National Institutes of Health, Bethesda, MD). Corneal scraping and microbial cultures were performed for patients who presented with corneal ulcers. Based on the results of the slit-lamp examination, TK cases were classified into the four subgroups: SPK (10 eyes; 18.5%), PDK (14 eyes; 25.9%), UK (16 eyes; 29.6%), and RK (14 eyes; 25.9%).

### In Vivo Confocal Microscopic Examination

For all subjects, IVCM images were obtained using the new Rostock Cornea Module for the Heidelberg Retina Tomograph (HRT III; Heidelberg Engineering, Heidelberg, Germany). The focus was adjusted by means of the live image controlled by a charge-coupled device color camera (640 × 480 pixels, RGB, 15 frames/s). Prior to the examination, a drop of topical anesthetic (proparacaine hydrochloride, 0.5%) was instilled into the lower conjunctival fornix. IVCM was performed by two experienced ophthalmologists (L.W. and Y.Z.). First, the section mode was applied to obtain IVCM images of the epithelium, stroma, and endothelium from the central corneal area (4 mm, pupil area) and four standard quadrants of the peripheral cornea (superior, inferior, nasal, and temporal, approximately 4–8 mm). Fifty high-quality images were acquired in each quadrant. Then, a minimum of three volume scans were performed to obtain images of the basal cells close to the basal lamina in every quadrant. Finally, six sequence scans were obtained with particular focus on the subbasal nerve plexus and epithelial DCs.

### Image Analysis

Three representative images of the basal cells, subbasal nerves, and DCs were selected by a masked observer (G.S.) and reviewed by two experienced masked IVCM specialists (L.W. and Z.W.). The definitions of each evaluation parameter are provided in Table 1. The results of each parameter were reported as the mean of the quantitative results from three images. Basal cells were defined as the deepest epithelial cells adjacent to Bowman's layer. Their density (cells/mm^2^) was calculated with the proprietary software within the HRT3 Rostock Cornea Module (RCM) (Heidelberg Engineering) in the manual mode. In addition, the level of reflectivity in the corneal stroma of all TK eyes was evaluated at depths in increments of 50 µm, from 100 µm to 450 µm. The grading standard (0–4) has been previously described.^23^ For grade 0, the nuclei of stromal cells were clearly defined; for grade 1, the keratocyte nuclei were visible with some background haze; for grade 2, some stromal cell nuclei were visible; for grade 3, stromal cells were almost completely obscured; and for grade 4, all stromal cells were completely obscured.

**Table 1.** Definition of IVCM Parameters for Basal Cell, Dendritiform Cell, Nerve Tortuosity, and Keratocyte Reflectivity

Parameters	Definition	Expressed in
Basal cell density	Number of basal epithelial cells counted within a frame/0.16 mm^2^	Cells/mm^2^
Dendritiform cell density	Number of dendritiform cells counted within a frame/0.16 mm^2^	Cells/mm^2^
Dendritiform cell size	Total area covered by the dendritiform cell	µm^2^
Corneal nerve fiber length	Total length of the subbasal nerve within a frame/0.16 mm^2^	µm/mm^2^
Nerve tortuosity	Calculated by LC = 1 – L/C	Arbitrary unit
Keratocyte reflectivity	Classified according to a semiquantitative scale	Arbitrary unit (0–4)

LC is the ratio of arc length to chord length; L is the linear distance between the two nerve ends, and C is the actual chord length between the two nerve ends.

DCs were chosen and observed at depths of 50 to 70 µm at the level of the basal epithelial layer, basal lamina, or subbasal nerves. DC size was defined as the total hyperreflective area, including the cell body and dendritic structures.^20^ The size of all DCs in a frame was manually annotated using ImageJ software. DC density was counted manually and expressed as cells/mm^2^.

Corneal nerve fiber (CNF) length was quantitatively analyzed as the total length of all of the nerve structures in a frame and divided by the frame area, including nerve main trunks and branches, using NeuroJ software, a semi-automated tracing program (http://www.imagescience.org/meijering/software/neuronj/).^24^ If the central corneal epithelium was absent, the CNF length near the ulcer and the center of cornea was measured. Considering the low CNF density in TK patients and to decrease measurement errors, the longest nerve in the IVCM frame was measured, and the value of three images was taken to calculate the nerve tortuosity. The nerve tortuosity was evaluated by the ratio of arc length to chord length (LC) with the following formula: LC = 1 – L/C, where L and C represent the linear distance and actual length between the two ends of the nerve, respectively.^25^

### Statistical Analysis

Statistical analysis was performed using R 3.6.1 (R Foundation for Statistical Computing, Vienna, Austria). For categorical variables, frequency and percentage should be used for statistical description. The Shapiro test was used to determine the normality of variables. Values are reported as the mean ± standard deviation or median and interquartile range. Intraclass correlation coefficients were calculated to assess the reliability of measurements between two independent observers. Fisher's exact test was used to compare categorical variables among groups. A generalized mixed-effects linear model was used to adjust the correlation of binocular data and compare continuous variables among disease groups and the control group. The significance level was 0.05 and was adjusted according to the Bonferroni criteria when multiple comparisons were performed. Spearman's correlation analysis was performed to address the correlation between corneal fluorescein staining or topical eye drop use and DC or subbasal nerve parameters.

## Results

### Patient Characteristics

Fifty-four eyes of 46 patients with TK and 20 eyes of 20 normal controls were included. The mean ages were 51.2 ± 13.2 years (range, 13–77) in the TK group and 49.7 ± 5.2 years (range, 39–57) in the control group (*P* = 0.96). There was no significant sex difference between the control and TK groups (*P* = 0.70). In the TK group, the average duration of topical medications was 3.1 ± 2.1 months, and the type and frequency of topical medications were 4.6 ± 1.6 types/subject and 26.9 ± 9.5 times/day, respectively. Three patients had received subconjunctival injections, and one patient had used topical ophthalmic anesthetics for 1 month.

**Figure 1.**
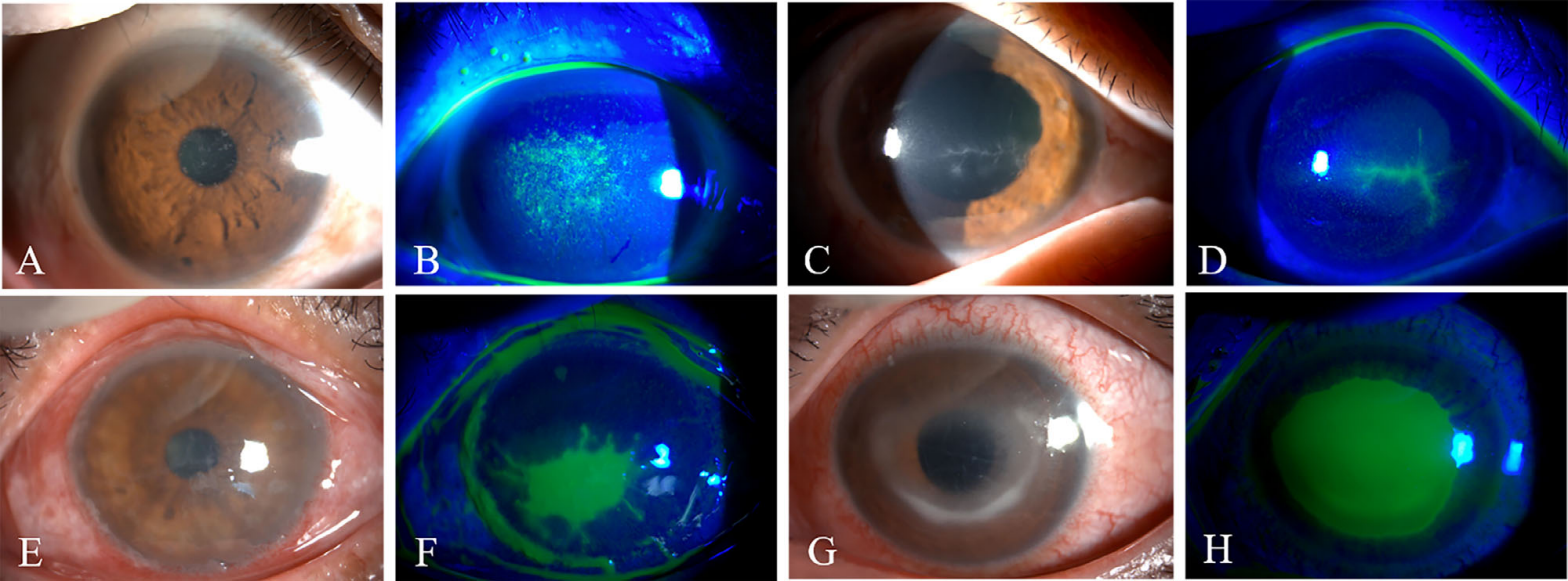
Slit-lamp photographs (white light and cobalt blue light) of toxic keratopathy. (**A**, **B**) Slit-lamp photographs of superficial punctate keratitis. (**C**, **D**) Pseudodendritic keratitis. (**E**, **F**) Ulcerative keratitis. (**G**, **H**) Ring keratitis.

The initial diagnoses included conjunctivitis (eight eyes, 14.8%), keratitis (35 eyes, 64.8%), iritis (four eyes, 7.4%), and postoperative complications (seven eyes, 13.0%) (Table 2). The typical clinical signs are shown in Figure 1. The majority of the corneal lesions were located in the inferior region (40 eyes, 74.1%) and central cornea (35 eyes, 64.8%), followed by the nasal (29 eyes, 53.7%), temporal (23 eyes, 42.6%), and superior (seven eyes, 13.0%) regions.

**Table 2.** Demographics and Clinical Characteristics of Toxic Keratopathy Patients and Controls

Parameter	TK Group	Control Group	*P*
Eyes, *n*	54	20	n/a
Age (y), mean ± SD	51.2 ± 13.2	49.7 ± 5.2	0.96
Sex (male/female), *n*	31/15	12/8	0.70
Topical medication history (mo), mean ± SD	3.1 ± 2.1	n/a	n/a
Number of topical medications, mean ± SD	4.6 ± 1.6	n/a	n/a
Frequency of topical medications (times per day), mean ± SD	26.9 ± 9.5	n/a	n/a
Primary diagnosis, *n* (%)			
Conjunctivitis	8 (14.8)	n/a	n/a
Keratitis	35 (64.8)	n/a	n/a
Iritis	4 (7.4)	n/a	n/a
Postoperative complication, *n* (%)	7 (13.0)	n/a	n/a
Location of lesion, *n* (%)			
Center	35 (64.8)	n/a	n/a
Superior	7 (13.0)	n/a	n/a
Inferior	40 (74.1)	n/a	n/a
Nasal	29 (53.7)	n/a	n/a
Temporal	23 (42.6)	n/a	n/a
Clinical manifestation, *n* (%)			
SPK	10 (18.5)	n/a	n/a
PDK	14 (25.9)	n/a	n/a
UK	16 (29.6)	n/a	n/a
RK	14 (25.9)	n/a	n/a

n/a, not applicable.

### In Vivo Confocal Microscopic Findings

**Figure 2.**
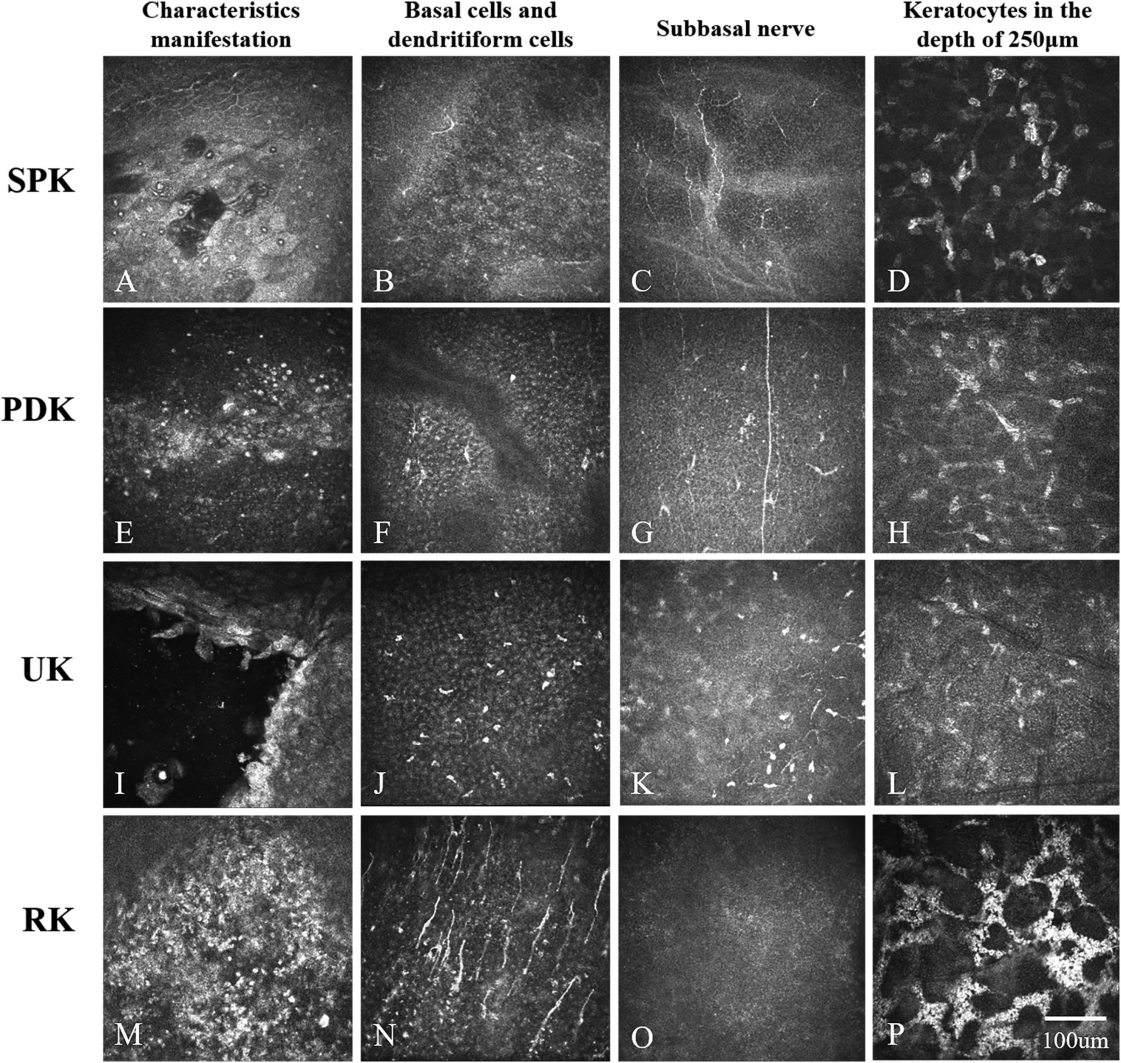
Representative in vivo confocal microscopic images of toxic keratopathy patients. The *left column* shows the characteristic manifestations of each subgroup, the *middle left column* shows basal cells and dendritiform cells, the *middle right column* shows an image frame of the subbasal nerves, and the *right column* shows keratocytes at a depth of 250 µm. (**A**) A dark zone and irregular, hyperreflective superficial cells are observed in SPK. (**E**) Hyperreflective linear lesions of PDK are seen. (**I**) The margin of an ulcer is clear with a hyperreflective edge in UK. (**M**) Anterior chamber cells are observed in RK.

With the quantitative measurements described above, the results of IVCM measurement were highly consistent (<5% variation) between the two independent observers. In the SPK group, irregular, hyperreflective superficial cells and dark acellular zones were observed in the epithelial layer (Fig. 2A). The IVCM characteristics of PDK cases included highly reflective twig-like structures, branches, and margins with a pseudo-dendritic appearance (Fig. 2E). In the UK group, the margins of the ulcers were clear with hyperreflective edges composed of edematous epithelial cells (Fig. 2I). A large infiltration of DC cells was also observed in patients with RK, with hyperreflective keratic precipitates (Fig. 2M). As shown in the right middle column of Figure 2, the subbasal nerves were slender, and their density was significantly decreased in each subgroup of TK. The keratocyte nuclei were ambiguous, and the volume of keratocytes increased along with the background haze. As the scan progressed in depth, keratocyte reflectivity decreased, and the nuclei were more distinguishable. The keratocyte reflectivity in each subgroup of TK showed noticeable differences in the same scanning layer (Fig. 2, right column). Corneal basal cells and DCs in each subgroup are shown in the left middle column.

### Basal Cells and Keratocytes

The mean basal cell density of the TK group was 43.1% less than that of the control group: 4103 cells/mm^2^ (range, 3331–4841) versus 7211 cells/mm^2^ (range, 7106–7632; *P* < 0.001) (Table 3). Further subgroup analysis indicated that the basal cell densities of the SPK (4432 cells/mm^2^; range, 3528–5356), PDK (4943 cells/mm^2^; range, 4584–5358), UK (3983 cells/mm^2^; range, 3187–4778), and RK (3494 cells/mm^2^; range, 3063–3861) groups were all significantly different from controls (all *P* < 0.001) (Supplementary Table). In addition, the difference in basal cell density between the PDK and RK groups was statistically significant (*P* = 0.002).

**Table 3.** Quantitative Parameters in TK Subjects and Controls

	TK Group	Control Group	Statistics	*P*
Eyes, *n*	54	20	—	—
Basal cell density (cells/mm^2^), median (IQR)	4103 (3331–4841)	7211 (7106–7632)	8.71	<0.001
DC density (cells/mm^2^), median (IQR)	45.8 (25.0–100.0)	24.0 (20.8–32.3)	–2.57	0.013
DC size (µm^2^), median (IQR)	111.0 (92.0–137.8)	63.7 (47.7–70.3)	–5.45	<0.001
CNF length (µm/mm^2^), median (IQR)	4051 (2844–5395)	19,905 (18,296–21,467)	17.59	<0.001
Nerve tortuosity, median (IQR)	0.08 (0.05–0.09)	0.04 (0.02–0.04)	–4.89	<0.001

IQR, interquartile range.

**Figure 3.**
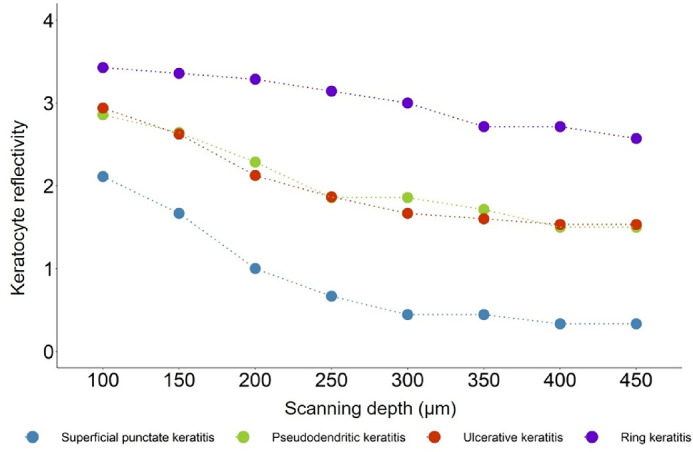
Keratocyte reflectivity changes with scan depth in four subtypes of toxic keratopathy.

Most of the TK cases showed significantly higher stromal keratocyte reflectivity than the control group (Fig. 3), whereas patients with SPK showed normal reflectivity in the stroma deeper than 250 µm. As scanning depth increased, the degree of keratocyte reflectivity decreased in each subgroup. The PDK and UK groups showed similar levels of hyperreflectivity (*P* = 0.777), which were higher than in the SPK group and lower than in the RK group (all *P* < 0.001).

### Dendritiform Cells

**Figure 4.**
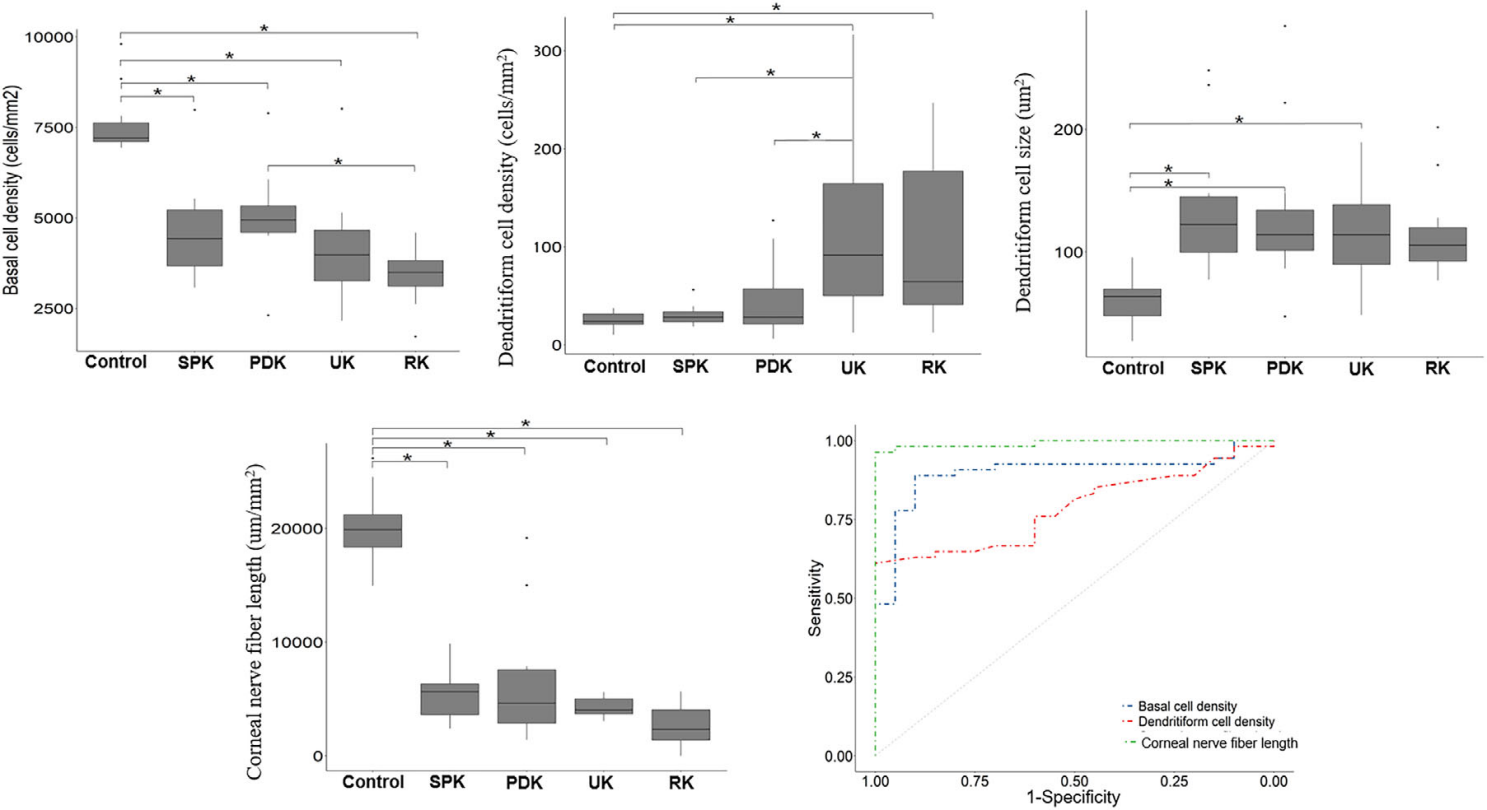
Changes in basal epithelial cell density, dendritiform cell density, dendritiform cell size, and corneal nerve fiber length in toxic keratopathy subjects and controls. ^*^Statistically significant adjusted *P* < 0.005.

Quantitative analysis of DC density and DC size in the TK and control groups is shown in Table 3 and Figure 4. The DC density of the TK group was significantly higher (45.8 cells/mm^2^; range, 25.0–100.0) than that of the control group (24.0 cells/mm^2^; range, 20.8–32.3) (*P* = 0.013). Interestingly, DC density in the UK and RK groups showed a statistically significant increase (91.7 cells/mm^2^; range, 50.0–164.6; *P* = 0.003; 64.6 cells/mm^2^; range, 40.6–193.8; *P* < 0.001) compared with normal controls (24.0 cells/mm^2^; range, 20.8–32.3; *P* < 0.001), whereas DC density was not increased in the SPK group (28.1 cells/mm^2^; range, 22.9–35.4; *P* = 0.481) or the PDK group (28.1 cells/mm^2^; range, 20.8–58.3; *P* = 0.095).

A statistically significant increase in DC size was detected in the total TK group (111.0 µm^2^; range, 92.0–137.8) compared with controls (63.7 µm^2^; range, 47.7–70.3 (*P* < 0.001). Further subgroup analysis showed that, compared with controls, DC size increased in all four TK subgroups, and statistically significant differences were found in the SPK group (122.5 µm^2^; range, 94.1–148.0; *P* < 0.001), PDK group (114.2 µm^2^; range, 100.8–135.4; *P* < 0.001), and UK group (114.2 µm^2^; range, 89.9–138.7; *P* = 0.001), but not in the RK group (105.6 µm^2^; range, 89.2–128.0), compared with 63.7 µm^2^ (range, 47.7–70.3; *P* = 0.009) in the control group. Moreover, no statistical difference was found between any two TK subgroups (all *P* ˃ 0.05) (Fig. 4).

### Subbasal Nerves

The TK group showed a significant reduction in CNF length (4051 µm/mm^2^; range, 2844–5395) compared with the control group (19,905 µm/mm^2^; range, 18,295–21,467; *P* < 0.001) (Table 3). In addition, there was a downward trend in CNF length in the SPK (5653 µm/mm^2^; range, 3468–6353), PDK (4643 µm/mm^2^; range, 2844–7847), UK (4051 µm/mm^2^; range, 3686–5056), and RK (2323 µm/mm^2^; range, 1246–4415) groups. CNF length in each TK subgroup showed statistically significant differences compared with the control group (all *P* < 0.001), but no statistical significance was found in pairwise comparison of the four TK subgroups. Meanwhile, nerve tortuosity was significantly different between the TK group (0.08; range, 0.05–0.09) and controls (0.04; range, 0.02–0.04) (*P* < 0.001) and among all four TK subgroups and controls (all *P* < 0.005). However, no statistically significant difference in nerve tortuosity was found (all *P* ˃ 0.05) among the four TK subgroups (Fig. 4).

When comparing the total TK group and controls, receiver operating characteristic (ROC) curves revealed that the area under the curve (AUC) for basal cell density was 0.899; for DC density, the AUC was 0.786; and for CNF length, the AUC was 0.992 (Fig. 4). The AUC appeared to indicate a more obvious discrimination power for CNF length compared with basal cell density or DC density. Additionally, with CNF length of 12426 µm/mm^2^, the sensitivity was 1.000 and the specificity was 0.963.

### Correlation Analysis

A Spearman's correlation was conducted to correlate the clinical parameters, DC parameters, and nerve parameters. The DC size was significantly correlated to the duration and frequency of topical eye drops (*r* = 0.361, *P* < 0.05; *r* = 0.581, *P* < 0.05), DC density (*r* = 0.247; *P* < 0.05), and nerve tortuosity (*r* = 0.417; *P* < 0.005). The increases in DC size and DC density both showed negative correlations to CNF length (*r* = −0.493; *P* < 0.005; *r* = −0.325; *P* < 0.005). In addition, the inverse correlation between CNF length and nerve tortuosity was significant (*r* = −0.666; *P* < 0.005) (Table 4).

**Table 4.** Correlations Among Corneal Fluorescein Staining, DC Density, DC Size, CNF Length, and Nerve Tortuosity

	Oxford Scale	Corneal Ulcer Size	DC Density	DC Size	CNF Length	Nerve Tortuosity
Oxford scale	1	—	—	—	—	—
Corneal ulcer size	n/a	1	—	—	—	—
DC density	0.181	0.225	1	—	—	—
DC size	–0.046	0.035	0.247^a^	1	—	—
CNF length	–0.238	–0.048	–0.325^b^	–0.493^b^	1	—
Nerve tortuosity	0.316	0.202	0.125	0.417^b^	–0.666^b^	1

a Statistically significant correlation coefficients (*P* < 0.05); Spearman's correlation analysis was used.

b Statistically significant correlation coefficients (*P* < 0.005).

## Discussion

In our study, IVCM findings in TK were as follows: Activated DC infiltration, subbasal nerve degeneration, and keratocyte reflectivity decreased with increasing depth. The quantitative results demonstrated an increase in DC density, DC size, and nerve tortuosity in the TK group, as well as a decrease in basal cell density and CNF length. Interestingly, further subgroup analysis revealed that CNF length in every subgroup decreased significantly; however, DC density was only statistically significantly higher in the UK and RK groups. A ROC curve showed that CNF length has the strongest discrimination power for TK. Thus, corneal IVCM characteristics may provide information for differential diagnosis and treatment selection in TK.

It is worth noting that 14.8% of the TK cases were diagnosed as conjunctivitis at their first visit and eventually developed keratitis, reflecting that caution in prescribing medication is paramount. Postoperative complications account for 13.0% of TK cases. A combination of prophylactic antibiotics, non-steroidal antiinflammatory drugs, and topical steroids was generally used after eye surgery, which also put patients at risk of TK, especially those with dry eye, diabetes, or rheumatic disease.^26^^,^^27^ It is helpful to avoid TK by evaluating the ocular surface, using postoperative medications appropriately, and monitoring the status of the cornea frequently.

In the present study, basal cell density decreased as the TK worsened, and the mean basal cell density decreased by 43.1% in TK participants, which is greater than the 31.0% seen in a limbal stem cell deficiency (LSCD) study with IVCM.^28^ The concurrence of LSCD might explain the delayed epithelial healing in some TK patients. It has been shown that several eye drops have damaging effects on limbal stem cells.^29^^–^^31^ A murine LSCD model has been induced by the application of topical benzalkonium.^32^ However, there are still very few human studies concentrating on LSCD caused by frequent topical medications and its prognosis. Furthermore, basal cell density in viral keratitis did not change significantly,^33^ which may potentially differentiate it from TK, but this requires more comparative study.

TK involves not only the corneal epithelial layer but also the stromal cells. The results show that PDK, UK, and RK cases have increased keratocyte reflectivity, suggesting an ongoing inflammatory reaction and a state of metabolic activation.^34^ The role of inflammation induced by topical eye drops on the cornea is confusing. Baghdasaryan et al.^21^ concluded that DC density increased significantly in patients with topical anti-glaucoma therapy; however, a previous study suggested that there was only a small, insignificant difference between unpreserved tafluprost and preserved latanoprost.^35^ Others have also found that DC density was not significantly different between normal controls and toxic SPK.^7^ Considering that most previous results had small sample sizes and did not conduct subgroup analyses, we assume that the different conclusions might be related to different stages and/or severities of TK being included in the various studies. Of note is that our results showed that DC size increased significantly in TK patients and had a positive correlation with the duration and frequency of topical drug use, and DC density in the total TK patient group was statistically different from controls; however, subgroup analysis revealed that the differences were not present in the SPK and PDK groups, but only in the UK and RK groups. These results show that preservative-free lubricants alone might be effective for patients with SPK or PDK. For toxic UK and RK, however, an immunologically mediated reaction was involved, and moderate antiinflammatory treatment was necessary.

Corneal nerves are critical for regulation of corneal sensation, maintenance of epithelial integrity and immune homeostasis, and corneal wound healing.^36^^,^^37^ CNF length drastically decreased and nerve tortuosity increased significantly, indicating that a large loss of subbasal nerves occurs and is accompanied by nerve regeneration in TK subjects.^38^ It is well established that TK and neurotrophic keratitis show similar slit-lamp presentations.^39^^,^^40^ However, there are still some differences. The affected area is more extensive and likely to be inferior or inferonasal, where medications gravitate, often accompanied by obvious conjunctival changes.^6^ Moreover, the drug deposition detected by IVCM was a direct indicator of TK. One thing to note is that significant reduction in CNF length was found even in patients with mild keratopathy in TK (71.6% for SPK), but DC density was only significantly increased in the more severe groups (UK and RK). DC density and DC size also exhibited a negative correlation with CNF length. This is a reminder that the changes in subbasal nerves occur in an earlier stage of TK and might precede immune responses. Whether early intervention in nerve growth is necessary may become clearer in the future with a better understanding of neurological rehabilitation and the inflammatory response in TK patients.

Interestingly, we found that there was no correlation between corneal fluorescein staining and IVCM parameters, which signifies that the clinical manifestation was not necessarily consistent with changes at the cellular level and might explain why healing time can be significantly different among TK patients. Wilson^6^ reported that some drug reactions did improve within 2 or 3 days, but the average period was 11 or 12 days, and the healing time ranged from 1 week to 3 months. Hence, the severity of the clinical manifestation was not an actual indicator of prognosis of TK patients. The loss of subbasal nerves, the inflammatory response, the dysfunction or deficiency of limbal stem cells, and other cytological changes may be the determining factors of recovery from TK and require further follow-up investigation.

Our study has some limitations. First, our study had a relatively small sample size for the TK subgroups, and underlying microstructural changes caused by medical management could have been included in these cohorts of patients. Second, considering the economic factors and informed consent of patients, laboratory tests to confirm the presence or absence of viruses were not conducted. Third, this was a cross-sectional study. Longitudinal studies are required to increase our understanding of the pathophysiology of TK and choose appropriate treatments.

IVCM images are able to show decreases in basal cell density and CNF length and increases in DC density, DC size, nerve tortuosity, and keratocyte reflectivity among TK patients. Such clinical observations might be significant for gaining an in-depth understanding of changes at the cellular level in TK. The specific fundamental mechanisms behind TK, as well as potential optimization of clinical interventions, should be the subject of further study.

## Supplementary Material

Supplement 1
