# Supplementary material for: Characteristics of Toxic Keratopathy, an In Vivo Confocal Microscopy Study
Source: Transl Vis Sci Technol. 2021 Sep 8;10(11):11. doi: 10.1167/tvst.10.11.11 (PMC8431974; doi:10.1167/tvst.10.11.11)
Supplement: Supplement 1 [file tvst-10-11-11_s001.pdf]

Supplementary Table: Quantitative parameters in SPK, PK, UK and RK. Values are expressed as median and interquartile range.

	Controls	SPK	PK	UK	RK	*p* value	Subgroup comparison
**Eyes** (n)	20	10	14	16	14	n/a	n/a
**BC** density (cells/mm2)	7211 (7106-7632)	4432 (3528-5356)	4943 (4584-5358)	3983 (3187-4778)	3494 (3063-3861)	<0.001	1, 2, 3, 4, 7
**DC density** (cells/mm2)	24.0 (20.8-32.3)	28.1 (22.9-35.4)	28.1 (20.8-58.3)	91.7 (50.0-164.6)	64.6 (40.6-193.8)	0.001	3, 4, 5
**DC size** (µm2)	63.7 (47.7-70.3)	122.5 (94.1-148.0)	114.2 (100.8-135.4)	114.2 (89.9-138.7)	105.6 (89.2-128.0)	<0.001	1, 2, 3, 4,
**CNF length** (µm/mm2)	19905 (18295-21467)	5653 (3468-6353)	4643 (2844-7847)	4051 (3686-5056)	2323 (1246-4415)	<0.001	1, 2, 3, 4, 6
**Nerve tortuosity**	0.04 (0.02-0.04)	0.07 (0.05-0.08)	0.07 (0.05-0.10)	0.06 (0.05-0.09)	0.09 (0.06-0.13)	<0.001	1, 2, 3, 4

Note: *P* values of Fisher exact test performed among the TK groups and controls; In Subgroup comparison, 1. controls vs. SPK; 2. controls vs. PDK; 3. controls vs. UK; 4. controls vs. RK; 5. SPK vs. UK; 6. SPK vs. RK; 7. PDK vs. RK. (*P*<0.005) (SPK: superficial punctate keratitis; PDK: pseudodendritic keratitis; UK: ulcerative keratitis; RK: ring keratitis; BC: basal cell; DC: dendritiform cell; CNF: corneal nerve fiber; n/a: not applicable).
